# MUC21 is downregulated in oral squamous cell carcinoma and associated with poor prognosis

**DOI:** 10.3389/fonc.2026.1767261

**Published:** 2026-03-25

**Authors:** Lisha Mao, Jia Kang, Anna Zou, Xiangpu Wang, Siyuan Guo, Sijia Chen, Ying Su, Lihua Ge, Jing Yang, Xuejiu Wang

**Affiliations:** 1 Department of Oral and Maxillofacial Plastic and Trauma Surgery, Capital Medical University School of Stomatology, Beijing, China; 2 Guang’anmen Hospital, China Academy of Chinese Medical Sciences, Beijing, China; 3 State Key Laboratory of Oral & Maxillofacial Reconstruction and Regeneration, National Clinical Research Center for Oral Disease, The Fourth Military Medical University, Xi’an, China; 4 Shaanxi Key Laboratory of Stomatology, Department of Oral Biology & Clinic of Oral Rare Diseases and Genetic Diseases, School of Stomatology, Xi’an, China; 5 Beijing Institute of Dental Research, Capital Medical University School of Stomatology, Beijing, China

**Keywords:** biomarker, biomarker analysis, MUC21, oral squamous cell carcinoma, prognosis

## Abstract

**Background:**

Although mucins are generally linked to aggressive tumor behavior and poor clinical outcomes, the role of MUC21 in oral squamous cell carcinoma (OSCC) remains unexplored. Previous *in vitro* studies suggest that MUC21 may inhibit cancer cell adhesion, indicating its potential significance in OSCC pathogenesis.

**Methods:**

We analyzed microarray data from 10 OSCC tissues and paired adjacent normal tissues (para-OSCC), along with GEO and TCGA RNA-seq datasets, to identify differentially expressed genes, including MUC21. qRT-PCR was performed to validate MUC21 expression and its co-expressed epithelial differentiation-related genes. Additionally, immunohistochemistry (IHC) on 102 paired OSCC/para-OSCC samples assessed the correlation between MUC21 expression and clinical outcomes. *In vitro* functional assays (CCK-8, wound healing, Transwell) were conducted using OSCC cell lines (CAL27, HN6) with MUC21 overexpression or knockdown.

**Results:**

MUC21 was significantly downregulated in OSCC compared to normal tissue, supported by high-throughput datasets, qRT-PCR, and IHC. We identified 11 co-expressed genes, including epithelial differentiation markers (KRT4, KRT13, CRNN), which were further validated. Low MUC21 expression correlated with pathological lymph node metastasis, poor tumor differentiation, and reduced survival. Furthermore, MUC21 Knockdown significantly increased cell proliferation, migration and invasion in OSCC cell lines and vice versa.

**Conclusion:**

MUC21 downregulation is associated with reduced epithelial differentiation, increased clinical aggressiveness, and worse prognosis in OSCC. MUC21 may serve as a novel prognostic biomarker and tumor suppressor gene in OSCC.

## 1 Introduction

Human mucins are categorized into membrane-bound types (MUC1, MUC3A, MUC3B, MUC4, MUC12, MUC13, MUC14, MUC15, MUC16, MUC17, MUC20, MUC21, MUC22) and secreted forms (MUC2, MUC5AC, MUC5B, MUC6, MUC7, MUC8, MUC9, MUC19), based on their structural properties (1). Physiologically, mucins are crucial for mucosal lubrication and protection, epithelial renewal, differentiation, and cellular signaling (2). In oncology, mucins significantly influence tumor genesis and progression across various cancers. Abnormal or deregulated mucin expression is associated with enhanced cancer cell proliferation, differentiation, migration, and invasion (3). For instances, MUC1 is overexpressed in pancreatic ductal adenocarcinoma and cervical squamous cell carcinoma and promotes cancer cell proliferation and metastasis (4, 5). MUC4 plays a vital role in esophageal squamous cell carcinoma cell proliferation and invasion (6) MUC16 is overexpressed in epithelial tumors like ovarian cancer and lung adenocarcinoma and facilitates increased tumor cell migration (7, 8).

Oral squamous cell carcinoma (OSCC) is associated with significant morbidity and a high mortality rate. Despite advances in surgical techniques, radiotherapy, and chemotherapy, the 5-year survival rate for OSCC remains around 60% (9). This is partly due to the absence of reliable prognostic indicators that could help clinicians devise effective primary treatment strategies, as the traditional TNM staging system has shown limited utility in this context (10). The molecular events underlying the pathogenesis and progression of OSCC contribute to its heterogeneity and are potential sources of diagnostic markers and therapeutic targets (11). Recent studies have identified several mucin members, such as MUC1 and MUC4, as being implicated in OSCC. These mucins are associated with more aggressive tumor behavior and poorer clinical outcomes (12).

MUC21 was first identified in 2008 as the human equivalent of mouse epiglycanin, a mucin expressed in mammary carcinoma TA3-Ha cells but absent in TA3-St cells (13). The MUC21 protein is characterized by an N-terminal signal sequence, an extracellular mucin domain containing dozens of tandem repeats 15-amino acid each, a stem domain, a transmembrane domain, and a C-terminal cytoplasmic tail (14). Increased expression of MUC21 has been observed to contribute to the anti-adhesion effects of cancer cells *in vitro*. Variations in MUC21 expression have been noted in several types of malignant tumors (15). A Previous study has documented overexpression of MUC21 in gingival inflammation (16). However, the role of MUC21 in OSCC has not yet been explored. The aim of this study is to delve deeper into the potential involvement of MUC21 in OSCC.

In this study, we first analyzed cDNA microarray data from our own research, combined with datasets from the Gene Expression Omnibus (GEO) and The Cancer Genome Atlas (TCGA), which revealed a significant downregulation of MUC21 and several of its co-expressed genes in OSCC. Next, we validated the altered expression of MUC21 and its epithelial differentiation-related co-expressed genes using quantitative real-time PCR (qRT-PCR) and immunohistochemistry (IHC). Furthermore, we assessed the correlation between MUC21 expression patterns and key clinical characteristics, including patient outcomes. Finally, we explored the functional impact of MUC21 in OSCC by conducting gain-of-function (overexpression) and loss-of-function (knockdown) experiments in OSCC cell lines. Our findings highlight MUC21 as a potential novel prognostic biomarker for OSCC.

## 2 Materials and methods

### 2.1 Patients and tissue samples

In this study, we initially performed microarray analysis on 10 paired samples (from patient cohort 1) of OSCC and adjacent normal mucosa (para-OSCC). To validate the candidate genes identified from the microarray and additional datasets obtained from the Gene Expression Omnibus (GEO) and The Cancer Genome Atlas (TCGA), we further analyzed 30 paired OSCC and para-OSCC samples (from patient cohort 2) using qRT-PCR and IHC. To investigate the association between MUC21 expression and clinicopathological features, we conducted IHC analysis on a retrospective cohort of 102 OSCC patients (patient cohort 3, treated between 2009 and 2013) with complete follow-up data. The patient enrollment process was shown in the **Supplementary Figure 1**.

All tissue specimens (OSCC and para-OSCC) were collected during surgical resection and histologically confirmed by frozen section examination prior to microarray profiling, qRT-PCR, and IHC studies. This study was approved by the Medical Ethics Committee of Capital Medical University School of Stomatology (Approval No. CMUSH-IRB-KJ-PJ-2018-04), and written informed consent was obtained from all participants in accordance with ethical guidelines.

### 2.2 Differential expressed genes’ analysis between OSCC and normal oral tissues based on our microarray data and datasets downloaded from GEO and TCGA

To perform microarray hybridization, total RNA was extracted from samples of various groups. RNA labeling and microarray hybridization were carried out following the protocols outlined in the Affymetrix Expression Analysis Technical Manual (Biotechnology Company, Shanghai, China). The microarrays were scanned using the GeneChip Scanner 3000 system (Affymetrix, Santa Clara, CA, USA) with the default settings on Command Console software 3.1 (Affymetrix). In addition, GSE34105 was downloaded from GEO data base (https://www.ncbi.nlm.nih.gov/geo/query/acc.cgi?acc=GSE34105), which comprised 62 OSCC and 16 normal oral tissue. Moreover, RNAseq data of 266 OSCC and 19 normal oral tissue was downloaded from the website of National Cancer Institute (https://portal.gdc.cancer.gov). The background correction and normalization of the three datasets raw data were performed using the Robust Multichip Average (RMA) algorithm. The Limma software package in the R programming language was utilized to identify differentially expressed genes, considering a fold change of >2 and an adj-P-value of <0.05 as statistically significant. Visualizations included a volcano plot created with ggplot2 (version 3.1.0) and a heatmap generated using the pheatmap package (version 1.0.12) in R. Furthermore, intersections of differential expressed genes (up-regulated and down-regulated) revealed by the three datasets were created by on the line tool Evenn (17).

### 2.3 Co expression analysis of MUC21

To identify genes related with MUC21, cor.test of R language was run in the TCGA based dataset to carry out Pearson correlation analysis of MUC21 and other genes. Genes with R value more than 0.7 and P value less than 0.001 were sorted out. R language package ggplot2, ggpubr and ggExtra were used to create the correlation analysis maps; circlize and corrplot were used to generate the circos map.

### 2.4 RNA extraction and quantitative qRT-PCR

Tissue samples were rapidly dip-frozen in liquid nitrogen and homogenized using an MX-F Vortex mixer (SCILOGEX, LLC, USA) before immediately preceding to RNA extraction. Total RNA was extracted from the homogenized frozen tissue using Trizol reagent (Invitrogen), adhering to the manufacturer’s protocol. Subsequently, 500 ng of RNA was reverse-transcribed into cDNA utilizing HiScript II Reverse Transcriptase (Vazyme, Nanjing, China). For quantitative real-time PCR (qRT-PCR), specific primers were used as follows: *MUC21* forward: GGGGCTCTTTGCTGGGCTCTT; *MUC21* reverse: CCGCTGTTCCTCCCGCTCAT. *KRT4* forward: CGCGAACAGATCAAGCTCCT; *KRT4* reverse: GGGGCTCAAGGTTTTTGCTG. *KRT13* forward: CCCCAGGCATTGACCTGAC; *KRT13* reverse: GTGTTGGTAGACACCTCCTTG. *CRNN* forward: ATGCCTCAGTTACTGCAAAACA; *CRNN* reverse: TCACATCGGCAAACTCTTGCT. *GAPDH* forward: TCAAGAAGGTGGTGAAGCAGG; *GAPDH* reverse: GCGTCAAAGGTGGAGGAGTG. The data were expressed as mean ± standard deviation (S.D.). GAPDH was utilized as the internal control. All assays were conducted in triplicate using the Roche LightCycler 96 System. Relative gene expression levels were calculated using the 2–ΔΔCT method.

### 2.5 Immunohistochemistry and semi-quantification of the results

For IHC analysis, 4 µm-thick paraffin sections of tissue samples were prepared. The IHC procedure followed previously established protocols (18). Initially, sections were deparaffinized and rehydrated. Endogenous peroxidase activity was quenched with a 3% hydrogen peroxide solution. Following antigen retrieval, sections were blocked with 10% normal serum and then incubated with either anti-MUC21 antibody (NBP2-31023; Polyclonal; 1:200; NOVUS Biologicals, Littleton, CO, USA), anti-KRT4 antibody (CY5773, monoclonal; 1:200; Abways Technology, Shanghai, China), anti-KRT13 antibody (CY5744, monoclonal; 1:100; Abways Technology, Shanghai, China) or anti-CRNN antibody (TA811824S, monoclonal; 1:500; OriGene Technologies Inc, Rockville, MD, US)for 2 hours at 37°C. This was followed by incubation with a secondary antibody, and color development was achieved using horseradish peroxidase enzyme and diaminobenzidine (DAB) chromogen reagent. Positive immunostaining was identified by cytoplasmic and/or membrane immunoreactivity. The intensities of the immunohistochemical reactions were evaluated under a light microscope (BX-71; Olympus, Japan) by three independent trained observers who were blinded to the subjects’ clinical information. Quantification of MUC21 or KRT4, KRT13 and CRNN expression levels was conducted using a computerized image analysis system (Image-Pro Plus V6.0, Media Cybernetics Inc, Bethesda, MD, USA). Five images at low-power magnification (×200) were captured randomly from each slide using a digital camera (DP72; Olympus, Japan), focusing on areas containing squamous epithelial carcinoma cells or normal squamous epithelial cells. Staining intensity was measured by mean optical density (MOD), corresponding to the positive staining intensity of MUC21. Expression levels were classified into high and low expression groups based on the median MOD value.

### 2.6 Survival analysis of MUC21 expression and other key clinical pathological factors

Clinicopathological data, including gender, age, tumor (T) classification, node (N) classification, and the overall tumor, node, metastases (TNM) stage, were extracted from clinical records and pathology reports. The TNM classification was based on the 7^th^ edition of the American Joint Committee on Cancer Staging System for oral cancer. During the follow-up period, overall survival (OS) was measured from the date of surgery until the date of the last follow-up examination or the patient’s death. Patients who were still alive at the time of analysis were censored at the date of their last follow-up. Disease-free survival (DFS) was defined as the time from the date of surgery to the date of disease recurrence/metastasis or death from any cause, whichever occurred first. Patients who were alive and free of recurrence/metastasis at the time of analysis were censored at the date of their last disease assessment. The clinicopathological variables, including the expression of MUC21, were evaluated in the analysis of survival (both OS and DFS) using the Kaplan-Meier method, providing a statistical approach to estimate the survival probability over time, taking into account various patient and disease characteristics.

### 2.7 Cell culture, lentiviral vector construction and transfection into OSCC cell lines

Human OSCC cell lines Cal27 and HN6 were acquired from the American Type Culture Collection (ATCC, USA). These cells were cultured in Dulbecco’s Modified Eagle Medium (DMEM, Gibco, USA). The culture medium was supplemented with 10% fetal bovine serum (FBS, Gibco, USA), 100 IU/mL penicillin, and 100 µg/mL streptomycin (Solarbio, China). In order to overexpress MUC21 in OSCC cell, Vector GV492 (GeneChem, Shanghai China) was used to construct MUC21 lentiviral expression system (LV-MUC21 lentivirus, NM_001010909; GeneChem, Shanghai China). To achieve knockdown of MUC21 in OSCC cells, the siRNA sequence was designed, synthesized and integrated into lentivirus (GeneChem, Shanghai China) targeting the specific region of the human MUC21 gene sequence, The targeted sequence of the siRNA was as follow: GTTTGGTCTACTATTGCATTT. The lentiviruses were transfected into Cal27 and HN6 using Lipofectamine 3000 reagent (Invitrogen, USA), following the manufacturer’s protocol.

### 2.8 Cell proliferation, wound healing, Transwell cell migration and invasion assays

Cell proliferation was assessed using a Cell Counting Kit-8 (CCK-8) (Solarbio, China) following the manufacturer’s instructions. Briefly, 3,000 OSCC cells were seeded into each well of a 96-well plate and incubated for three days. At designated time points, 10 µL of CCK-8 solution was added to each well and incubated at 37°C for 2 hours. Absorbance was then measured at 450 nm. An *in vitro* wound healing assay was employed to evaluate cell motility. Briefly, transfected OSCC cells (1 × 10^5^ cells/well) were seeded into 6-well plates. Upon reaching 90% confluence, wounds were created by dragging a 10 µl pipette tip across the well from one end to the other. The cells were then cultured for up to 24 hours. Wound closure was monitored under 100X magnification, and cell migration rates were calculated by comparing images taken at 0 and 24 hours. Transwell chambers (Corning, USA) equipped with 8-µm-pore polycarbonate filters coated with Matrigel(Corning, USA)or without were used to assess Cell invasion or migration. OSCC cells (2 × 10^5^ cells/ml) were suspended in DMEM supplemented with 0.1% FBS. Subsequently, 200 µl of this suspension was placed in the upper chamber, and 700 µl of DMEM supplemented with 10% FBS was added to the lower chamber. Following a 24-hour incubation period at 37°C with 5% CO2, cells that had migrated or invaded through the polycarbonate membrane were fixed with 4% formaldehyde for 10 minutes and subsequently with 100% methanol for 20 minutes. The fixed cells were then stained with crystal violet for 15 minutes. Cells remaining on the upper surface of the membrane were removed using a cotton swab. Six random microscopic fields at 100x magnification were photographed and analyzed for cell count. All experiments were conducted in triplicate.

### 2.9 Statistical analysis

Statistical analyses were performed using SPSS 22.0 and GraphPad Prism 8. For validation experiments, comparisons between paired OSCC and normal tissues in qRT-PCR and IHC data were analyzed using the Wilcoxon signed-rank test, and correlations were assessed with Spearman’s coefficient. Associations between MUC21 expression and clinicopathologic features were evaluated with the Chi-square or Fisher’s exact test. Survival outcomes were compared using Kaplan-Meier curves and the log-rank test. Prognostic factors were identified by univariate and multivariate Cox proportional hazards regression, with model assumptions verified (**Supplementary Figure 2**). For *in vitro* functional assays, two-tailed unpaired Student’s t test and one-way ANOVA were performed to evaluate significant differences. All P-values are two-sided, and significance was defined as P< 0.05.

## 3 Results

### 3.1 MUC21 expression decreased in OSCC and was related with KRT4, KRT13 and CRNN

**Figure 1:**
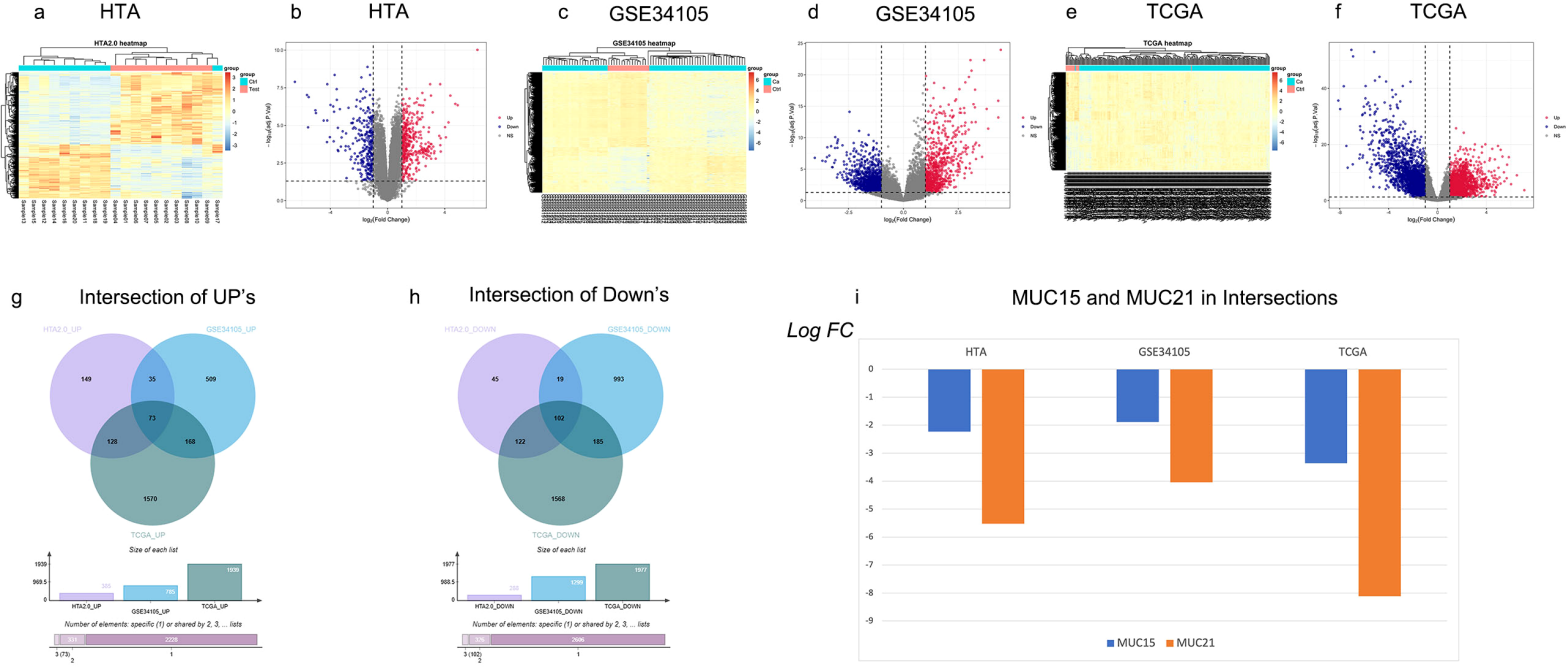
MUC21 Expression by analyzing high-throughput dataset including Affymetrix GeneChip^®^ Human Transcriptome Array 2.0(HTA), GSE34105 from GEO and TCGA RNAseq data. **(A-F)**, the heatmaps of hierarchical clustering and volcano plots of differential gene expressions revealed by HTA based on 10 paired OSCC and adjacent normal oral tissue; GSE34105 based on 62 OSCC and 16 normal oral tissue; TCGA base on 266 OSCC and 19 normal oral tissue. Genes with a fold change >2 and adj-P-value of <0.05 were highlighted. **(G, H)** the up and down regulated genes in the three datasets were intersected and it was showed that 73 was up and 102 were down regulated in the intersection which comprised only MUC15 and MUC21 from Mucin family. **(I)** relative expression level (the median of the three datasets) comparison showed that MUC21 was more down regulated than MUC15, the chart was created from Excel (Microsoft^®^ for Excel). OSCC, oral squamous cell carcinoma.

**Figure 2:**
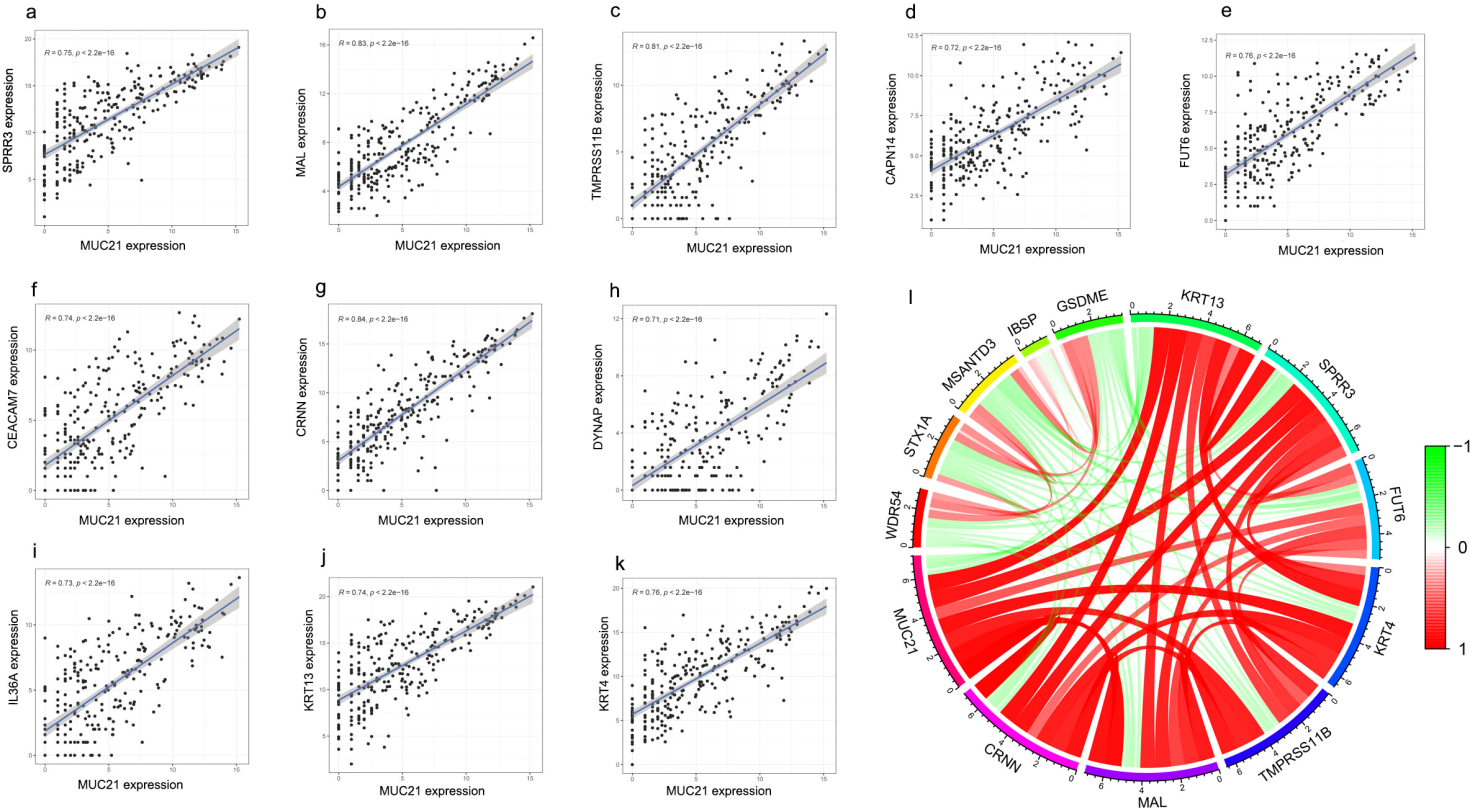
MUC21 co expression gene analysis by cor.test of R language in the TCGA. **(A-K)**, the correlation analysis of MUC21 to SPRR3, MAL, TMPRSS11B, CAPN14, FUT6, CEACAM7, CRNN, DYNAP, IL36A, KRT13, KRT4. R value was set at more than 0.7, and P value was set less than 0.001. **(L)**, Top five negatively or positively correlated genes were showed in circos map.

**Figure 3:**
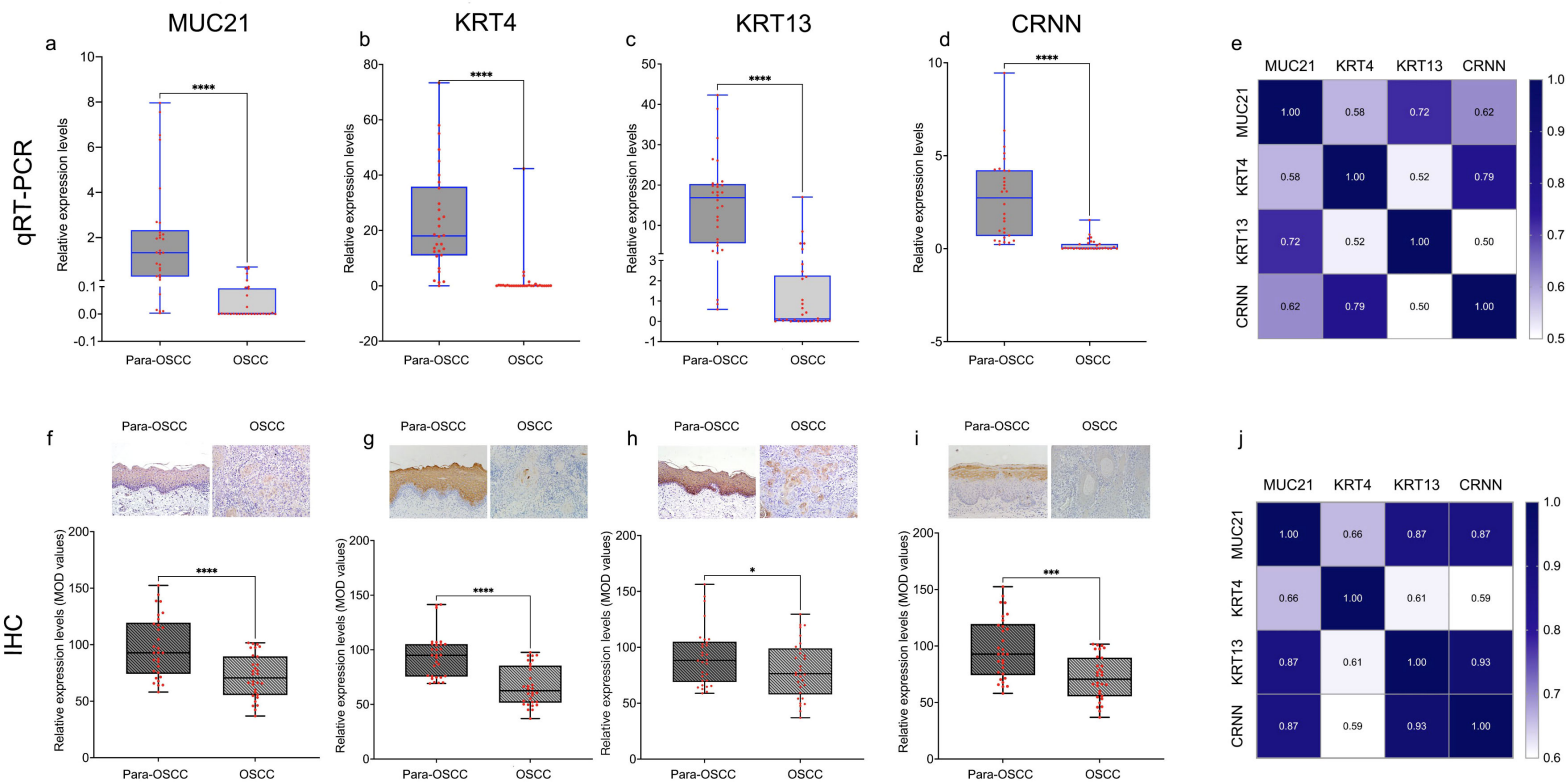
Quantitative qRT-PCR and immunohistochemistry analysis of MUC21, KRT4, KRT13, and CRNN in OSCC and para-OSCC. **(A-D)** showed that MUC21, KRT4, KRT13 and CRNN were down regulated in OSCC than para-OSCC (P< 0.0001), the expression levels were normalized against GAPDH. **(E)** Spearman correlation analysis showed that MUC21was related with KRT4, KRT13 and CRNN at mRNA level. **(F-I)** showed that MUC21 and KRT4 were down regulated in OSCC than para-OSCC (P<0.0001); KRT13 was down regulated in OSCC than para-OSCC (P <0.05); and CRNN was also down regulated in OSCC than para-OSCC (P<0.001). **(J)** Spearman correlation analysis showed that MUC21 was related with KRT4, KRT13 and CRNN at protein level too.

The intersection of HTA (our microarray dataset, **Figures 1A, B**), GSE34105(**Figures 1C, D**) and the dataset from TCGA (**Figures 1E, F**) showed that 73 genes were up regulated (**Figure 1G**) and 102 genes were down regulated (**Figure 1H**). The intersection gene list was presented in **Supplementary Table 1**. Among them, MUC15 and MUC21 from the Mucin family were identified, both of which were down regulated, with MUC21 showing the most significant decrease (**Figure 1I**). The Co-expression analysis of MUC21 with other genes revealed that 11 genes including KRT4, KRT13 and CRNN were closely related with MUC21 in expression levels (**Figure 2**). Differentiation-specific genes, keratin4/13 for non-keratinizing epithelia and keratin1/10 for keratinizing epithelia, are known to be expressed in pairs within the oral squamous cell epithelium (19). Besides, CRNN, also known as Cornulin, is related to the late epidermal differentiation. To further verify this finding, MUC21, KRT4, KRT13 and CRNN expression was assessed in additional 30 paired samples using qRT-PCR and IHC analysis. The results confirmed that both MUC21 and KRT4, KRT13, CRNN were downregulated in OSCC tissues (**Figure 3**). The Spearman rank correlation test showed that the expressions of MUC21, KRT4, KRT13 and CRNN were related. (**Figures 3E, J**).

### 3.2 Decreased MUC21 expression was associated with more clinical aggressiveness in OSCC

**Figure 4:**
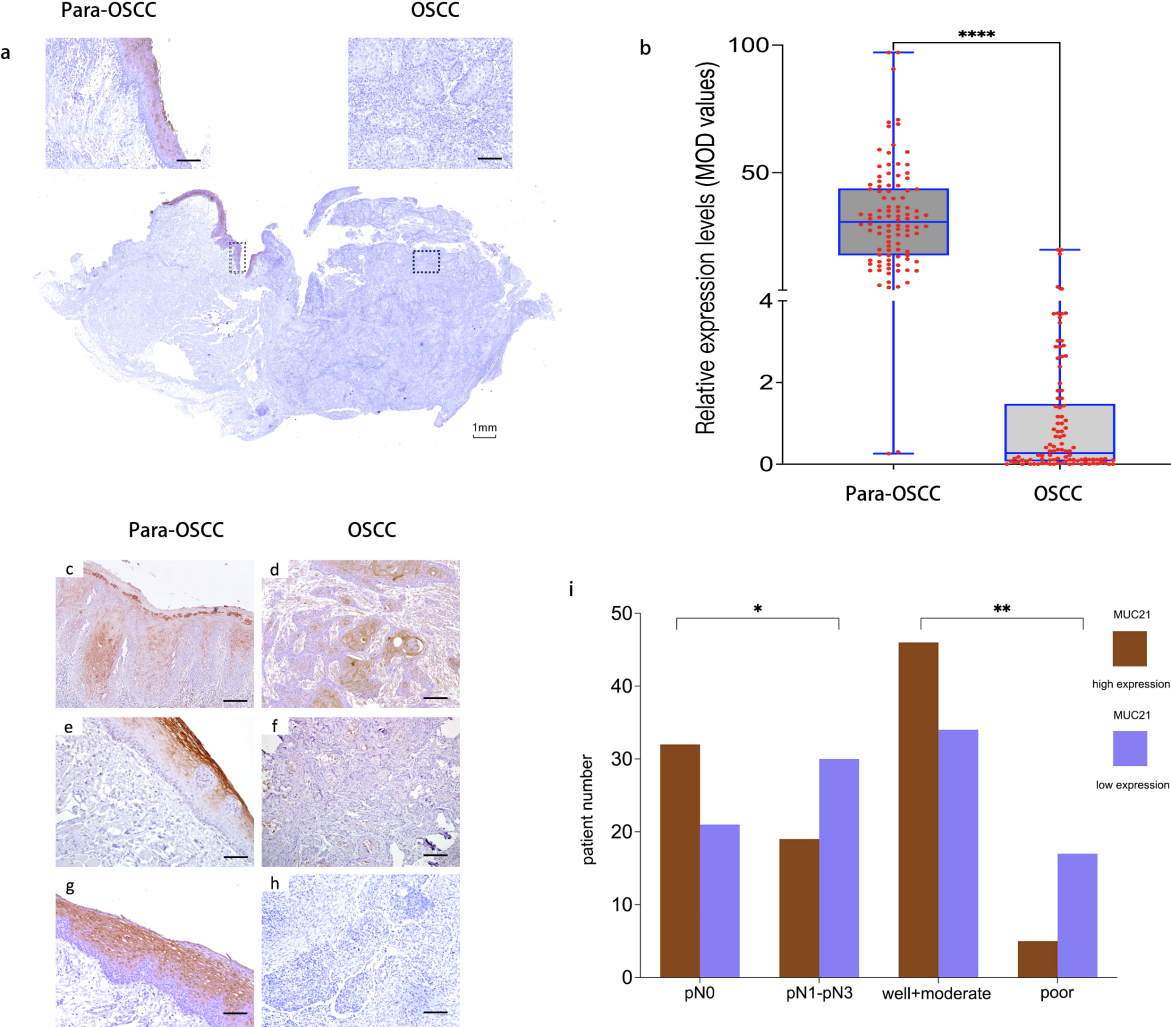
MUC21 expression analysis in OSCC and para-OSCC via immunohistochemistry (IHC) and its relation with critical clinical characters. **(A)** a whole block of OSCC and para-OSCC tissue analyzed by IHC showed that MUC21 was expressed in para-OSCC epithelium and lost in OSCC. **(B)** MUC21 expression between OSCC and para-OSCC in 102 paired patient samples was quantified by mean optical density (MOD) values. MUC21 decreased significantly in OSCC (P < 0.0001). Box plots represent the median, 25th, and 75th percentiles of the data. **(C–H)** displayed matched para-OSCC and OSCC. **(D, F, H)** represented well, moderate, and poor differentiation of OSCC, respectively. Unannotated Scale bar = 100 μm. The Scale bar of the block tissue was 1mm. **(I)** decreased MUC21 expression level was related with cervical lymphatic metastasis and OSCC differentiation. “pN0” means no lymphatic metastasis, “pN1-PN3” referred to different degrees of lymphatic metastasis; “well +moderate” and “poor” referred to different degrees of differentiation.

The IHC results of MUC21 showed that it was expressed in para-OSCC epithelium, and obviously decreased in OSCC (**Figures 4A, B**, P<0.0001). IHC analysis of MUC21 expression in 102 patients showed that the reduction of MUC21 was correlated with different degrees of tumor differentiation (**Figures 4C-H**). Analysis using the Chi-square test revealed significant associations between low MUC21 expression and several adverse clinicopathologic features. Specifically, the presence of pathological lymph node metastasis was more frequent in the low MUC21 expression group compared to the high expression group (P = 0.029). Similarly, poor tumor differentiation was significantly associated with low MUC21 expression (P = 0.01, **Figure 4I**). the corresponding detailed results and statistical analysis was also presented in **Supplementary Table 2**.

### 3.3 Decreased MUC21 expression was a prognostic factor for worse OS and DFS in OSCC

**Figure 5:**
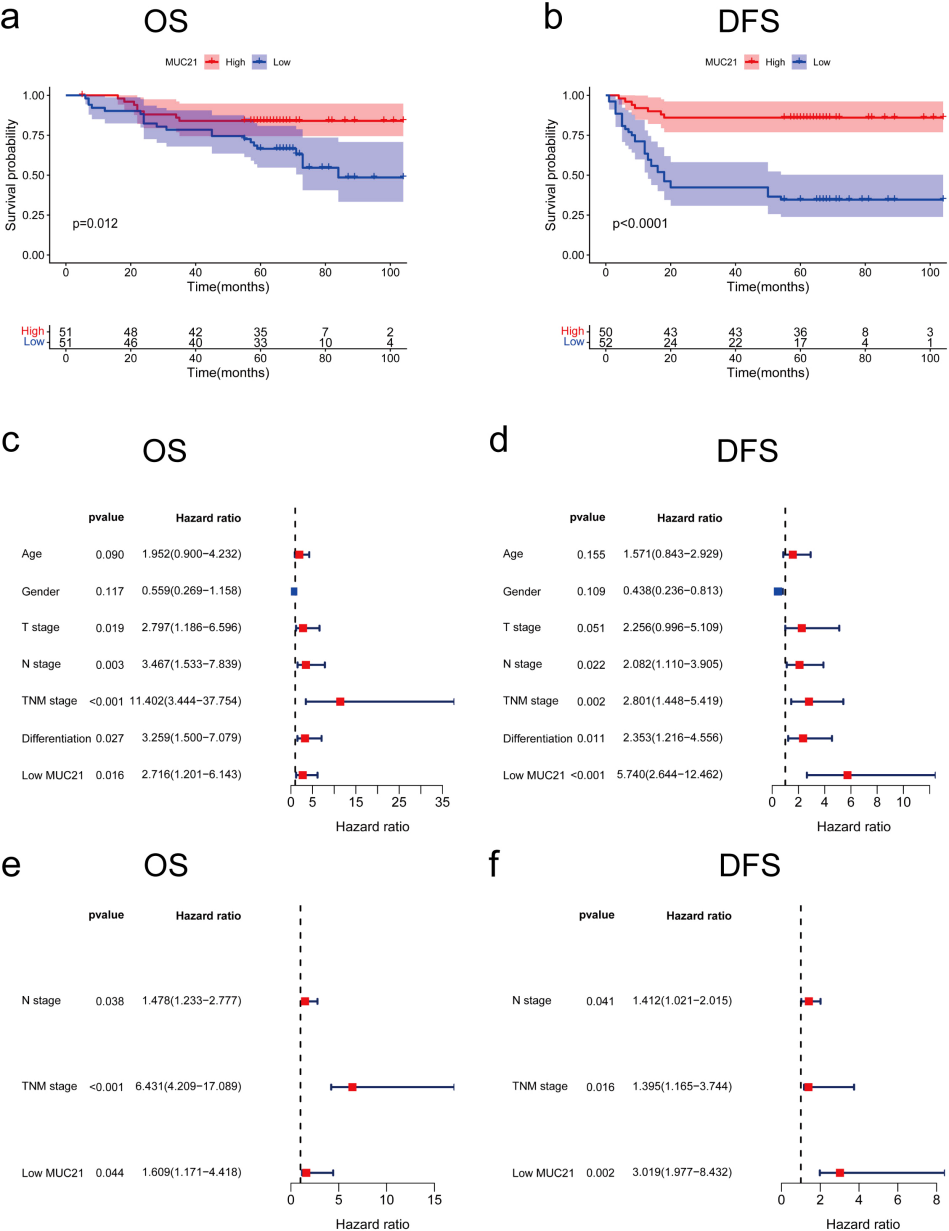
Kaplan–Meier survival analyses for postoperative OSCC patients based on MUC21 expression. **(A, B)** Overall Survival (OS) and disease-free survival (DFS)with low MUC21 expression were significantly shorter than those with high MUC21 expression (P = 0.012 for OS, P<0.0001 for DFS). **(C, D)**, Forest map: The univariate analysis of OS and DFS in OSCC patients. **(E, F)**, Forest map: The multivariate analysis of OS and DFS in OSCC patients.

We collected clinicopathological and follow-up data from 102 patients, including 29 OS deaths and 41 DFS events. The median follow-up time was 65 months (56.5-71). Kaplan-Meier survival analysis, supplemented by the log-rank test, was employed to assess the differences in OS and DFS among patients with OSCC based on MUC21 expression levels. The results indicated that patients in the low MUC21 expression group had significantly lower OS and DFS rates compared to those in the high expression group (**Figures 5A, B**). Similarly, reduced survival rates were also observed in patients with advanced TNM stages, presence of lymph node metastasis, and poor tumor differentiation (**Supplementary Figure 3**). Further statistical analysis using Cox proportional hazards regression models in both univariate and multivariate contexts confirmed the significance of MUC21 expression as a prognostic indicator (**Figures 5C–F**). In the univariate analysis, low MUC21 expression was associated with a negative impact on OS (P = 0.044, **Table 1**) and was even more strongly correlated with reduced DFS (P = 0.002, **Table 2**).

**Table 1:** Univariate and multivariate analysis of overall survival in OSCC.

Variable	No. of patients(%)		Univariate analysis			Multivariate analysis		
	Alive	Dead	HR	95%CI	P-value	HR	95%CI	P-value
Age, y								
<60	41(56.2)	10(34.5)	1		0.090			
≥60	32(43.8)	19(65.5)	1.952	0.900-4.232				
Gender								
Female	26(35.6)	15(51.7)	1		0.117			
Male	47(64.4)	14(48.3)	0.559	0.000-1.158				
P- T classification								
T1 or T2	69(94.5)	22(75.9)	1		0.019			0.088
T3 or T4	4(5.5)	7(24.1)	2.797	1.186-6.596				
P-lymph node metastasis								
Negative (pN0)	50(68.5)	3(10.3)	1		0.003	1		0.038
Positive (pN1-pN3)	23(31.5)	26(89.7)	3.467	1.533-7.839		1.478	1.233-2.777	
TNM tumor stage								
I or II	48(65.8)	3(10.3)	1		<0.001	1		<0.001
III or IV	25(34.2)	26(89.7)	11.402	3.444-37.754		6.431	4.209-17.089	
Tumor differentiation								
Well or Moderate	64(87.7)	18(62.1)	1		0.027			0.069
Poor	9(12.3)	11(37.9)	3.259	1.500-7.079				
MUC21 expression								
High	43(58.9)	8(27.6)	1		0.016	1		
Low	30(41.1)	21(72.4)	2.716	1.201-6.143		1.609	1.171-4.418	0.044

The impact of clinical pathological factors along with MUC21 expression on the overall survival (OS)of 102 patients with oral squamous cell carcinoma (OSCC) was evaluated using Cox proportional hazards regression models. The univariate analysis identified tumor classification, pathologic lymph node metastasis, advanced TNM stage, poor tumor differentiation, and low MUC21 expression as significant negative prognostic factors. Subsequent multivariate analysis further confirmed pathologic lymph node metastasis, advanced TNM stage, and low MUC21 expression as independently significant negative prognostic factors for OS. Abbreviations: CI, confidence interval; HR, hazard ratio; OSCC, oral squamous cell carcinoma; P, pathological.

**Table 2:** Univariate and multivariate analysis of disease-free survival in OSCC.

Variable	No. of patients(%)		Univariate analysis			Multivariate analysis		
	Alive	Dead	HR	95%CI	P-value	HR	95%CI	P-value
Age, y								
<60	34(55.7)	17(41.5)	1		0.155			
>=60	27(44.3)	24(58.5)	1.571	0.843-2.929				
Gender								
Female	23(37.7)	18(43.9)	1		0.109			0.071
Male	38(62.3)	23(56.1)	0.438	0.236-0.813				
P-T classification								
T1 or T2	57(93.4)	34(82.9)	1		0.051			
T3 or T4	4(6.6)	7(17.1)	2.256	0.996-5.109				
P- lymph node metastasis								
Negative (pN0)	38(62.3)	16(39.0)	1		0.022	1		0.041
Positive (pN1-pN3)	23(37.7)	25(61.0)	2.082	1.110-3.905		1.412	1.021-2.015	
TNM tumor stage								
I or II	38(62.3)	13(31.7)	1		0.002	1		0.016
III or IV	23(37.7)	28(68.3)	2.801	1.448-5.419		1.395	1.165-3.744	
Tumor differentiation								
Well or Moderate	54(88.5)	28(68.3)	1		0.011			0.386
Poor	7(11.5)	13(31.7)	2.353	1.216-4.556				
MUC21 expression								
High	43(70.5)	8(19.5)	1		<0.001	1		0.002
Low	18(29.5)	33(80.5)	5.740	2.644-12.462		3.019	1.977-8.432	

The impact of clinical pathological factors along with MUC21 expression on the disease-free survival (DFS) of 102 patients with oral squamous cell carcinoma (OSCC) was evaluated using Cox proportional hazards regression models. The univariate analysis revealed that pathologic lymph node metastasis, advanced TNM stage, poor tumor differentiation, and low MUC21 expression significantly impacted DFS rate negatively. In the multivariate analysis, advanced TNM stage and MUC21 expression emerged as independent negative prognostic factors for DFS. Abbreviations: CI, confidence interval; HR, hazard ratio; OSCC, oral squamous cell carcinoma; P, pathological.

### 3.4 Overexpression of MUC21 inhibited and knockdown of it increased cell malignancy *in vitro*

**Figure 6:**
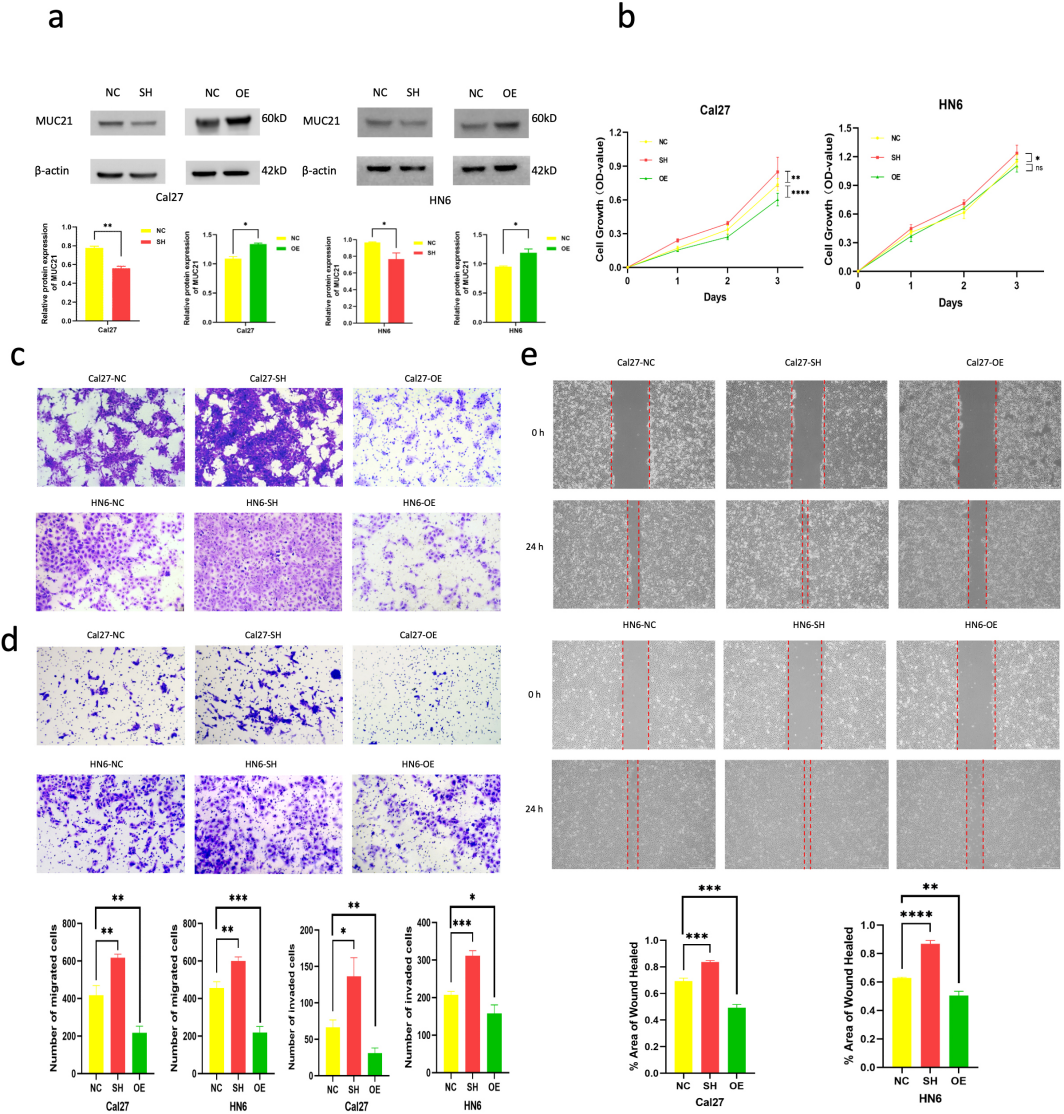
*In vitro* cell lines experiment post overexpression and knockdown of MUC21. **(A)** MUC21 was significantly overexpressed and knocked down in CAL27 and HN6. **(B)** CCK-8 assay on CAL27 and HN6 post MUC21 manipulation. **(C)** Transwell assay without Matrigel coated filter of CAL27 and HN6 post MUC21 manipulation. **(D)** Transwell assay with Matrigel coated filter of CAL27 and HN6 post MUC21 manipulation. **(E)** wound-healing assay conducted at 24 hours post MUC21 manipulation in CAL27 and HN6 cells. NC: negative control, SH: MUC21 knockdown, OE: MUC21 overexpression. Statistical significance is denoted by *P<0.05, **P<0.01, ***P<0.001, ****P<0.0001.

MUC21 was significantly overexpressed and knocked down in OSCC cell lines CAL27 and HN6 (**Figure 6A**). Cck-8 test showed that knockdown of MUC21 caused CAL27 and HN6 cell proliferation increased, yet MUC21 overexpression made CAL27 and HN6 less proliferative (**Figure 6B**). Transwell experiment showed that both CAL27 and HN6 migrated and invaded more violently when MUC21 was knocked down but changed to be less aggressive when MUC21 was over expressed in contrast to control (**Figures 6C, D**). Wound healing test further confirmed that less MUC21 increased cell migration and more MUC21 inhibited it both in CAL27 and HN6 (**Figure 6E**).

## 4 Discussion

In this study, we observed that the downregulation of MUC21 is a prevalent occurrence in OSCC. Furthermore, the extent of this reduction correlates with the aggressive characteristics of OSCC, including lymph node metastasis and poor clinical outcomes. In addition, downregulation of MUC21 in OSCC cell lines CAL27 and HN6 increased their malignancies and upregulation of MUC21 decreased their malignancies. These findings suggest that MUC21 may serve as a potential novel prognostic marker for OSCC.

Clinically the TNM staging system established by American joint committee on cancer has been and is now the main basis to appraise OSCC status and prognosticate its clinical outcome (20). But it still has been being constantly improved to raise its accuracy in appraising the malignancy of OSCC to which biomarkers might provide some extra help (21). Several dozens of biomarkers have been identified in OSCC to help predicting its clinical aggressiveness so as to choose the appropriate therapy regime or even develop the treatment targets, such as EGFR, PD-L1 (11, 22, 23). More specific biomarkers in OSCC are still on the way to be elucidated. Mucins are a family of highly glycosylated protein members involved in many gastro-intestinal cancers and other cancers (24–28). In OSCC, upregulation of MUC1 has been observed compared with normal oral mucosa (29) and it promotes OSCC invasion and migration through PI3K-Akt pathway (30). Silencing MUC1 inhibits OSCC proliferation invasion and migration and promotes its apoptosis at the same time (31). MUC4 has also been found upregulated in OSCC and is a risk factor for its prognosis (32). Elevated CA125(MUC16) in saliva has been found in OSCC patients and might be a prognostic marker (33). Bioinformatic analysis showed that MUC7 is one of hub gene (34). Increased MUC20 has been related with invasiveness of OSCC cells (35). However, to our knowledge, MUC21 has never been studied in OSCC before. Our results may be the first report about MUC21 in OSCC and provide new insight into the functions of mucins in OSCC.

In contrary to our result, several studies show that MUC21 is upregulated in cancer cells and promote cancer cell migration and metastasis. MUC21 can promote pancreatic cancer perineural invasion and metastasis (15). MUC21 expression was significantly upregulated in melanoma and promote melanoma cell lines’ proliferation and migration *in vitro* (36). In human glioblastoma tissues and cell lines, the elevated expression of MUC21 was observed and its high expression was related with glioblastoma cell viability and motility (37). MUC21 is also proved to inhibit both cytotoxic activity of NK cells and hinder T cell activation so that it severs as potent immunosuppressive factor in cancer (38). It has been shown by *in vitro* study that over expressed MUC21 in mouse cells decreased the adhesion among cells and cells to extracellular matrix components resulting in more floating, round cells during cell culture (39), and MUC21 transfection in to HEK293 cell decreased the number of apoptotic cells (40). However, in our study it was showed that MUC21 was expressed in non-tumor oral mucosa and its expression decreased in the corresponding squamous carcinoma cells both by microarray, bioinformatic, qRT-PCR and IHC methods. Furthermore, the invitro downregulation of MUC21 in OSCC cell lines CAL27 and HN6 increased their abilities of proliferation, migration and invasion, while up regulation of MUC21 inhibited those abilities which suggests that MUC21 is not a cancer cell metastasis enhancer in OSCC. This might seem contradictory to the previous reports. However, bioinformatic analysis has shown that MUC21 is downregulated in laryngeal squamous cell carcinoma (LSCC) (41). Furthermore, MUC21 has been found being expressed in opposite ways in malignant cells of different subtypes even in the same organ, it was expressed high in lung adenocarcinoma but low in squamous cell carcinoma (42). Besides, contradictory functions of both membrane-bound and secretory mucins have also been reported. For example, MUC16 have been shown to facilitate pancreatic cancer metastasis via FAK-mediated upregulation of mesenchymal markers (43), while depletion of MUC16 from the cell surface led to the internalization of E-cadherin, causing enhanced expression of mesenchymal markers vimentin and N-cadherin (44). Similar phenomenon has also been reported in MUC15 and MUC5AC (3, 45–47). Hence, the discrepancy of MUC21expression in different malignant cells might indicate that it works in different ways according to the cell types and scenarios.

The IHC and clinical findings from this study showed a significant correlation between the downregulation of MUC21 and low tumor differentiation in OSCC. This correlation is further supported by the close association of MUC21 expression levels with those of KRT4, KRT13 and CRNN, proteins specific to the differentiation of oral squamous epithelia. Notably, KRT4 and KRT13 have also been reported to be downregulated in OSCC (48, 49). Furthermore, downregulation of CRNN is an independent predictor of relapse in OSCC (50). In healthy oral tissues, both secreted and membrane-bound mucins play a critical role in protecting the epithelia by forming a mucous barrier on the surface of the apical epithelial cells (19). Though most membrane-bound mucins are upregulated and contribute to oncogenesis and malignancy development in cancers including OSCC, several of them, however, decreased in cancerous tissue than in matched non-cancerous tissue (51–53). In accordance with these findings, MUC21 was also downregulated in OSCC and related with cell differentiation, which in combination with both the clinical data and *in vitro* cell lines experiments results suggests that MUC21 is tumor suppressor gene in OSCC. It should be noted that our survival analysis was based on the AJCC 7th edition staging system. We acknowledged that key prognostic factors such as depth of invasion, which was formally incorporated in the 8th edition, should be considered in future assessments of biomarkers like MUC21. Further extensive and in-depth studies on MUC21 in OSCC and other malignancies are warranted to fully elucidate its precise role in tumor biology.

In summary, our study has meticulously analyzed the expression of MUC21 in OSCC and established that it is significantly downregulated in OSCC, relates with epithelial differentiation and correlates with more tumor aggressiveness and worse prognosis. Our results suggest that MUC21 could serve as a new marker in the prognosis of OSCC.

## 5 Conclusion

This study extensively explored MUC21 expression in OSCC by high through put dataset analysis, qRT-PCR and IHC in paired OSCC and para-OSCC, investigated *in vitro* behavior changes of OSCC cell lines when MUC21 was overexpressed or knocked down. It was confirmed that MUC21 was downregulated in OSCC. Through clinical investigation of 102 OSCC patients, it was found that MUC21 was related with more clinical aggressiveness and less survival rate. In contrary to the reported effect of MUC21 to cancer cells *in vitro*, our study showed that knockdown of MUC21 caused more aggressiveness in OSCC cell lines and overexpression of MUC21 made them more temperate *in vitro*. Our study indicates that MUC21 could serve as a new marker in the prognosis of OSCC and might be a tumor suppressor gene in OSCC.

## Data Availability

The datasets presented in this study can be found in online repositories. The data presented in the study are deposited in the NCBI-GEO repository, accession number GSE325374.

## Supplementary material

Supplementary Figure 1 The patient enrollment process.

Supplementary Figure 2 K-M curves of OS and DFS analysis of TNM stage, Lymph metastasis and Tumor differentiation.

Supplementary Figure 3 Assessment of proportional hazards assumption.

## References

[1] Gan GL Liu J Chen WJ Ye QQ Xu Y Wu HT . The diverse roles of the mucin gene cluster located on chromosome 11p15.5 in colorectal cancer. Front Cell Dev Biol. (2020) 8:514. doi: 10.3389/fcell.2020.00514 32695780 PMC7338833

[2] Sun L Zhang Y Li W Zhang J Zhang Y . Mucin glycans: A target for cancer therapy. Molecules. (2023) 28:7033. doi: 10.3390/molecules28207033 37894512 PMC10609567

[3] Chen T Chen Z Lian X Wu W Chu L Zhang S . MUC 15 promotes osteosarcoma cell proliferation, migration and invasion through Livin, MMP-2/MMP-9 and Wnt/β-catenin signal pathway. J Cancer. (2021) 12:467–73. doi: 10.7150/jca.49641 33391443 PMC7739004

[4] Bose M Grover P Sanders AJ Zhou R Ahmad M Shwartz S . Overexpression of MUC1 induces non-canonical TGF-beta signaling in pancreatic ductal adenocarcinoma. Front Cell Dev Biol. (2022) 10:821875. doi: 10.3389/fcell.2022.821875 35237602 PMC8883581

[5] Zhao A Pan Y Gao Y Zhi Z Lu H Dong B . MUC1 promotes cervical squamous cell carcinoma through ERK phosphorylation-mediated regulation of ITGA2/ITGA3. BMC Cancer. (2024) 24:559. doi: 10.1186/s12885-024-12314-6 38702644 PMC11069143

[6] Li S Liu Z Chen Q Chen Y Ji S . A novel fatty acid metabolism-related signature identifies MUC4 as a novel therapy target for esophageal squamous cell carcinoma. Sci Rep. (2024) 14:12476. doi: 10.1038/s41598-024-62917-z 38816411 PMC11139939

[7] Patel JS Callahan BM Chobrutskiy BI Blanck G . Matrix-metalloprotease resistant mucin-16 (MUC16) peptide mutants represent a worse lung adenocarcinoma outcome. Proteomics Clin Appl. (2019) 13:e1800155. doi: 10.1016/b978-0-323-55433-6.00006-7 30790454

[8] Schuster-Little N Sokolovsky AD Gentry A Saraf A Etzel MR Patankar MS . Immunoaffinity-free chromatographic purification of ovarian cancer biomarker CA125 (MUC16) from blood serum enables mass spectrometry characterization. Anal Methods. (2024) 16:6337–48. doi: 10.1039/d4ay01172d 39177265 PMC11342825

[9] Dong L Xue L Cheng W Tang J Ran J Li Y . Comprehensive survival analysis of oral squamous cell carcinoma patients undergoing initial radical surgery. BMC Oral Health. (2024) 24:919. doi: 10.1186/s12903-024-04690-z 39123139 PMC11313127

[10] Tehzeeb H Hande A Patil S Sonone A Pakhale A Chavhan A . Correlation of clinical and pathological TNM staging with histopathological grading in oral squamous cell carcinoma. Cureus. (2024) 16:e60912. doi: 10.7759/cureus.60912 38910661 PMC11193662

[11] Cheng Y Song Z Cheng J Tang Z . JARID2, a novel regulatory factor, promotes cell proliferation, migration, and invasion in oral squamous cell carcinoma. BMC Cancer. (2024) 24:793. doi: 10.1186/s12885-024-12457-6 38961353 PMC11220990

[12] Lan Z Zou KL Cui H Zhao YY Yu GT . Porphyromonas gingivalis suppresses oral squamous cell carcinoma progression by inhibiting MUC1 expression and remodeling the tumor microenvironment. Mol Oncol. (2024) 18:1174–88. doi: 10.1002/1878-0261.13517 37666495 PMC11076995

[13] Itoh Y Kamata-Sakurai M Denda-Nagai K Nagai S Tsuiji M Ishii-Schrade K . Identification and expression of human epiglycanin/MUC21: a novel transmembrane mucin. Glycobiology. (2008) 18:74–83. doi: 10.1093/glycob/cwm118 17977904

[14] Fini ME Jeong S Gong H Martinez-Carrasco R Laver NMV Hijikata M . Membrane-associated mucins of the ocular surface: New genes, new protein functions and new biological roles in human and mouse. Prog Retin Eye Res. (2020) 75:100777. doi: 10.1016/j.preteyeres.2019.100777 31493487 PMC10276350

[15] Chen Y Zhang W Zeng Y Yang P Li Y Liang X . GDNF-induced phosphorylation of MUC21 promotes pancreatic cancer perineural invasion and metastasis by activating RAC2 GTPase. Oncogene. (2024) 43:2564–77. doi: 10.1038/s41388-024-03102-4 39020072

[16] Bao X Wiehe R Dommisch H Schaefer AS . Entamoeba gingivalis causes oral inflammation and tissue destruction. J Dent Res. (2020) 99:561–7. doi: 10.1177/0022034520901738 32023135

[17] Yang M Chen T Liu YX Huang L . Visualizing set relationships: EVenn's comprehensive approach to Venn diagrams. Imeta. (2024) 3:e184. doi: 10.1002/imt2.184 38898979 PMC11183158

[18] Mao L Zhuang R Qin L Han Z Huang X Chen R . CCL18 overexpression predicts a worse prognosis in oral squamous cell carcinoma (OSCC). Neoplasma. (2020) 67:700–6. doi: 10.4149/neo_2020_190821n802 32202908

[19] Vaidya M Dmello C Mogre S . Utility of keratins as biomarkers for human oral precancer and cancer. Life (Basel). (2022) 12:343. doi: 10.3390/life12030343 35330094 PMC8950203

[20] Struckmeier AK Eichhorn P Agaimy A Buchbender M Moest T Lutz R . Comparison of the 7th and revised 8th UICC editions (2020) for oral squamous cell carcinoma: How does the reclassification impact staging and survival? Virchows Arch. (2024) 484:901–13. doi: 10.1007/s00428-023-03727-y 38191928 PMC11186894

[21] Subramaniam N Clark JR Goldstein D de Almeida J Abdalaty AHA Balasubramanian D . Geographical heterogeneity in the American Joint committee on Cancer oral cancer staging and prognostic implications. Oral Oncol. (2021) 113:105122. doi: 10.1016/j.oraloncology.2020.105122 33352532

[22] Jin N An Y Tian Y Zhang Z He K Chi C . Multispectral fluorescence imaging of EGFR and PD-L1 for precision detection of oral squamous cell carcinoma: a preclinical and clinical study. BMC Med. (2024) 22:342. doi: 10.1186/s12916-024-03559-w 39183296 PMC11346054

[23] Han Y Peng Y Xiong H Zeng L Zhang T Xia K . XPO1 serves as a prognostic marker involving AKT/MAPK/TGFBR1 pathway in OSCC. Cancer Med. (2024) 13:e70076. doi: 10.1002/cam4.70076 39177040 PMC11342079

[24] Balmaña M Duran A Gomes C Llop E López-Martos R Ortiz MR . Analysis of sialyl-Lewis x on MUC5AC and MUC1 mucins in pancreatic cancer tissues. Int J Biol Macromol. (2018) 112:33–45. doi: 10.1016/j.ijbiomac.2018.01.148, PMID: 29408556

[25] Li Z Yang D Guo T Lin M . Advances in MUC1-mediated breast cancer immunotherapy. Biomolecules. (2022) 12:952. doi: 10.3390/biom12070952 35883508 PMC9313386

[26] Ning Y Zheng H Zhan Y Liu S Yang Y Zang H . Comprehensive analysis of the mechanism and treatment significance of mucins in lung cancer. J Exp Clin Cancer Res. (2020) 39:162. doi: 10.1186/s13046-020-01662-3 32807223 PMC7433199

[27] Peng L Li Y Gu H Xiang L Xiong Y Wang R . Mucin 4 mutation is associated with tumor mutation burden and promotes antitumor immunity in colon cancer patients. Aging (Albany NY). (2021) 13:9043–55. doi: 10.18632/aging.202756 33714943 PMC8034916

[28] Sheng YH Davies JM Wang R Wong KY Giri R Yang Y . MUC1-mediated macrophage activation promotes colitis-associated colorectal cancer via activating the interleukin-6/ signal transducer and activator of transcription 3 axis. Cell Mol Gastroenterol Hepatol. (2022) 14:789–811. doi: 10.1016/j.jcmgh.2022.06.010 35809803 PMC9424590

[29] Abdelwhab A Shaker O Aggour RL . Expression of Mucin1 in saliva in oral squamous cell carcinoma and oral potentially Malignant disorders (case control study). Oral Dis. (2023) 29:1487–94. doi: 10.1111/odi.14138 35080082

[30] Li P Xiao LY Tan H . Muc-1 promotes migration and invasion of oral squamous cell carcinoma cells via PI3K-Akt signaling. Int J Clin Exp Pathol. (2015) 8:10365–74. PMC4637559 26617744

[31] Zhang AM Chi XH Bo ZQ Huang XF Zhang J . MUC1 gene silencing inhibits proliferation, invasion, and migration while promoting apoptosis of oral squamous cell carcinoma cells. Biosci Rep. (2019) 39:BSR20182193. doi: 10.1042/bsr20182193 31439759 PMC6747000

[32] Abidullah M Nahar P Ahmed SA Kothari H Vakeel S . MUC4 expression in oral dysplastic epithelium and oral squamous cell carcinoma: An immunohistochemical study. J Int Soc Prev Community Dent. (2023) 13:124–32. doi: 10.4103/jispcd.jispcd_241_22 37223448 PMC10202254

[33] Geng XF Du M Han JX Zhang M Tang XF Xing RD . Saliva CA125 and TPS levels in patients with oral squamous cell carcinoma. Int J Biol Markers. (2013) 28:216–20. doi: 10.5301/jbm.5000001 23613350

[34] Wu Q Cao R Chen J Xie X . Screening and identification of biomarkers associated with clinicopathological parameters and prognosis in oral squamous cell carcinoma. Exp Ther Med. (2019) 18:3579–87. doi: 10.3892/etm.2019.7998 31608128 PMC6778814

[35] Sasahira T Kurihara-Shimomura M Shimomura H Bosserhoff AK Kirita T . Identification of oral squamous cell carcinoma markers MUC2 and SPRR1B downstream of TANGO. J Cancer Res Clin Oncol. (2021) 147:1659–72. doi: 10.1007/s00432-021-03568-9 33620575 PMC11802022

[36] Liu X Xiao Y Xiong X Qi X . MUC21 controls melanoma progression via regulating SLITRK5 and hedgehog signaling pathway. Cell Biol Int. (2022) 46:1458–67. doi: 10.1002/cbin.11817 35579188

[37] Wang L Zhang X Liu J Liu Q . MUC21 induces the viability and migration of glioblastoma via the STAT3/AKT pathway. Exp Ther Med. (2022) 23:331. doi: 10.3892/etm.2022.11260 35401801 PMC8987941

[38] Lee DH Ahn H Sim HI Choi E Choi S Jo Y . A CRISPR activation screen identifies MUC-21 as critical for resistance to NK and T cell-mediated cytotoxicity. J Exp Clin Cancer Res. (2023) 42:272. doi: 10.1186/s13046-023-02840-9 37858248 PMC10588101

[39] Yi Y Kamata-Sakurai M Denda-Nagai K Itoh T Okada K Ishii-Schrade K . Mucin 21/epiglycanin modulates cell adhesion. J Biol Chem. (2010) 285:21233–40. doi: 10.1074/jbc.m109.082875 20388707 PMC2898422

[40] Tian Y Denda-Nagai K Tsukui T Ishii-Schrade KB Okada K Nishizono Y . Mucin 21 confers resistance to apoptosis in an O-glycosylation-dependent manner. Cell Death Discov. (2022) 8:194. doi: 10.1038/s41420-022-01006-4 35410995 PMC9001685

[41] Mo BY Li GS Huang SN He WY Xie LY Wei ZX . The underlying molecular mechanism and identification of transcription factor markers for laryngeal squamous cell carcinoma. Bioengineered. (2021) 12:208–24. doi: 10.1080/21655979.2020.1862527 33315534 PMC8291796

[42] Lin S Tian C Li J Liu B Ma T Chen K . Differential MUC22 expression by epigenetic alterations in human lung squamous cell carcinoma and adenocarcinoma. Oncol Rep. (2021) 45:78. doi: 10.3892/or.2021.8029 33786615 PMC8020203

[43] Muniyan S Haridas D Chugh S Rachagani S Lakshmanan I Gupta S . MUC16 contributes to the metastasis of pancreatic ductal adenocarcinoma through focal adhesion mediated signaling mechanism. Genes Cancer. (2016) 7:110–24. doi: 10.18632/genesandcancer.104 27382435 PMC4918949

[44] Comamala M Pinard M Theriault C Matte I Albert A Boivin M . Downregulation of cell surface CA125/MUC16 induces epithelial-to-mesenchymal transition and restores EGFR signalling in NIH : OVCAR3 ovarian carcinoma cells. Br J Cancer. (2011) 104:989–99. doi: 10.1038/bjc.2011.34 21326240 PMC3065274

[45] Han T Zheng H Zhang J Yang P Li H Cheng Z . Downregulation of MUC15 by miR-183-5p.1 promotes liver tumor-initiating cells properties and tumorigenesis via regulating c-MET/PI3K/AKT/SOX2 axis. Cell Death Dis. (2022) 13:200. doi: 10.1038/s41419-022-04652-9 35236826 PMC8891362

[46] Kesari MV Gaopande VL Joshi AR Babanagare SV Gogate BP Khadilkar AV . Immunohistochemical study of MUC1, MUC2 and MUC5AC in colorectal carcinoma and review of literature. Indian J Gastroenterol. (2015) 34:63–7. doi: 10.1007/s12664-015-0534-y 25731647

[47] Pothuraju R Rachagani S Krishn SR Chaudhary S Nimmakayala RK Siddiqui JA . Molecular implications of MUC5AC-CD44 axis in colorectal cancer progression and chemoresistance. Mol Cancer. (2020) 19:37. doi: 10.1186/s12943-020-01156-y 32098629 PMC7041280

[48] Li X Fang J Tao X Xia J Cheng B Wang Y . Splice site m(6)A methylation prevents binding of DGCR8 to suppress KRT4 pre-mRNA splicing in oral squamous cell carcinoma. PeerJ. (2023) 11:e14824. doi: 10.7717/peerj.14824 36811004 PMC9939020

[49] Naganuma K Hatta M Ikebe T Yamazaki J . Epigenetic alterations of the keratin 13 gene in oral squamous cell carcinoma. BMC Cancer. (2014) 14:988. doi: 10.1186/1471-2407-14-988 25527207 PMC4364656

[50] Govindaraj PK Kallarakkal TG Mohd Zain R Tilakaratne WM Lew HL . Expression of Ki-67, Cornulin and ISG15 in non-involved mucosal surgical margins as predictive markers for relapse in oral squamous cell carcinoma (OSCC). PloS One. (2021) 16:e0261575. doi: 10.1371/journal.pone.0261575 34941961 PMC8700009

[51] Matsuyama T Ishikawa T Mogushi K Yoshida T Iida S Uetake H . MUC12 mRNA expression is an independent marker of prognosis in stage II and stage III colorectal cancer. Int J Cancer. (2010) 127:2292–9. doi: 10.1002/ijc.25256 20162577

[52] Yang B Wu A Hu Y Tao C Wang JM Lu Y . Mucin 17 inhibits the progression of human gastric cancer by limiting inflammatory responses through a MYH9-p53-RhoA regulatory feedback loop. J Exp Clin Cancer Res. (2019) 38:283. doi: 10.1186/s13046-019-1279-8 31262330 PMC6604468

[53] Wang S Jin J Chen J Lou W . MUC14-related ncRNA-mRNA network in breast cancer. Genes (Basel). (2021) 12:1677. doi: 10.3390/genes12111677 34828282 PMC8620399

