# Supplementary figures and images for: MUC21 is downregulated in oral squamous cell carcinoma and associated with poor prognosis
Source: Front Oncol. 2026 Mar 25;16:1767261. doi: 10.3389/fonc.2026.1767261 (PMC13056625; doi:10.3389/fonc.2026.1767261)

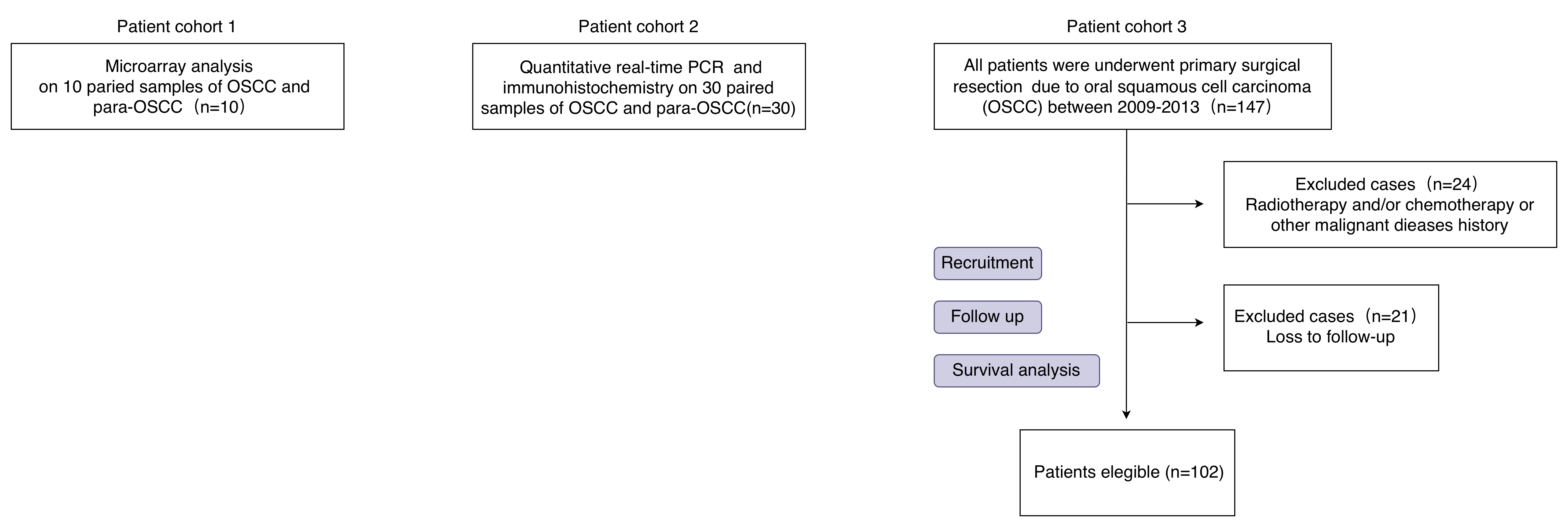

Supplement: Supplementary Figure 1 — The patient enrollment process. [file Image1.jpeg]

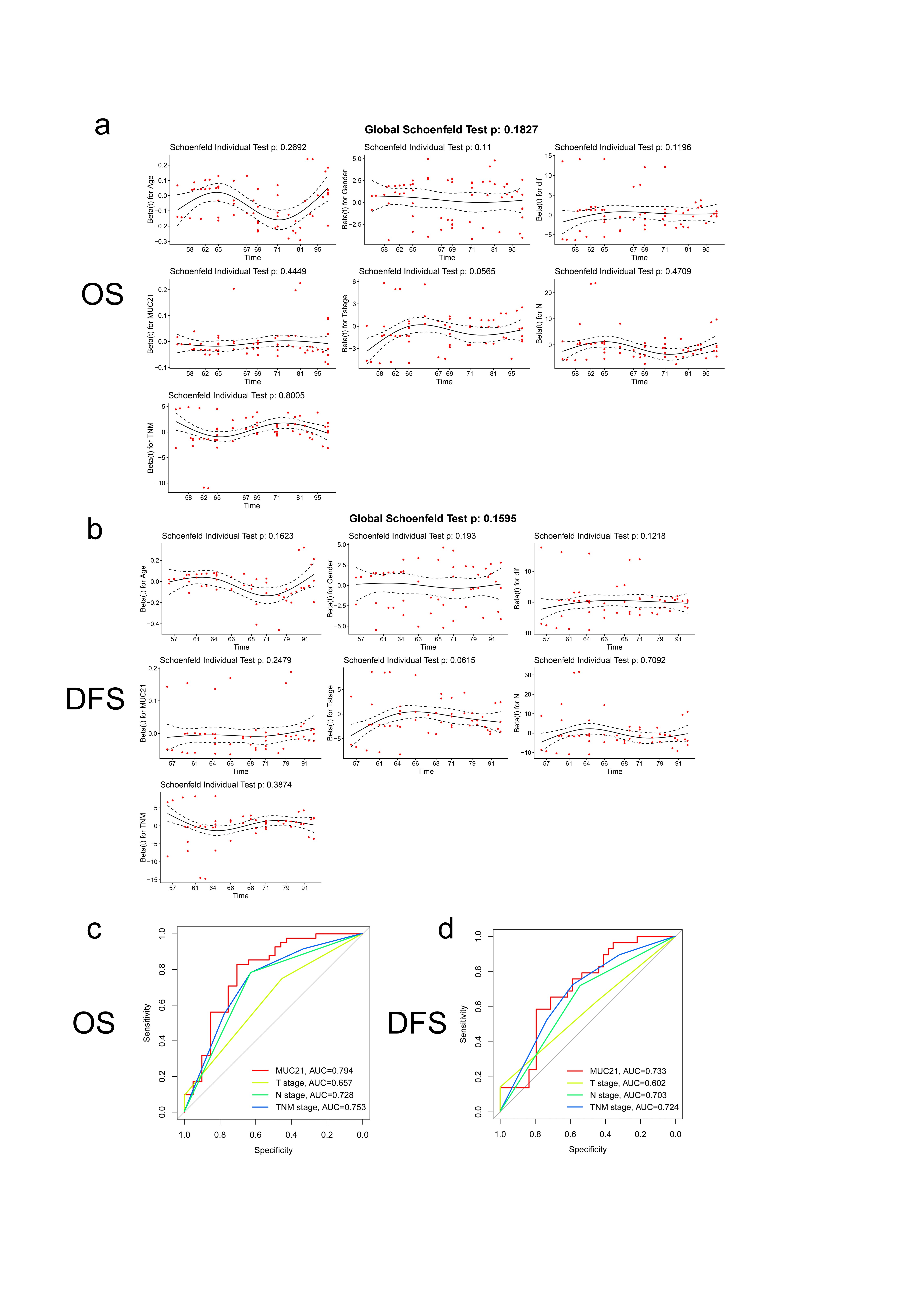

Supplement: Supplementary Figure 2 — K-M curves of OS and DFS analysis of TNM stage, Lymph metastasis and Tumor differentiation. [file Image2.jpeg]

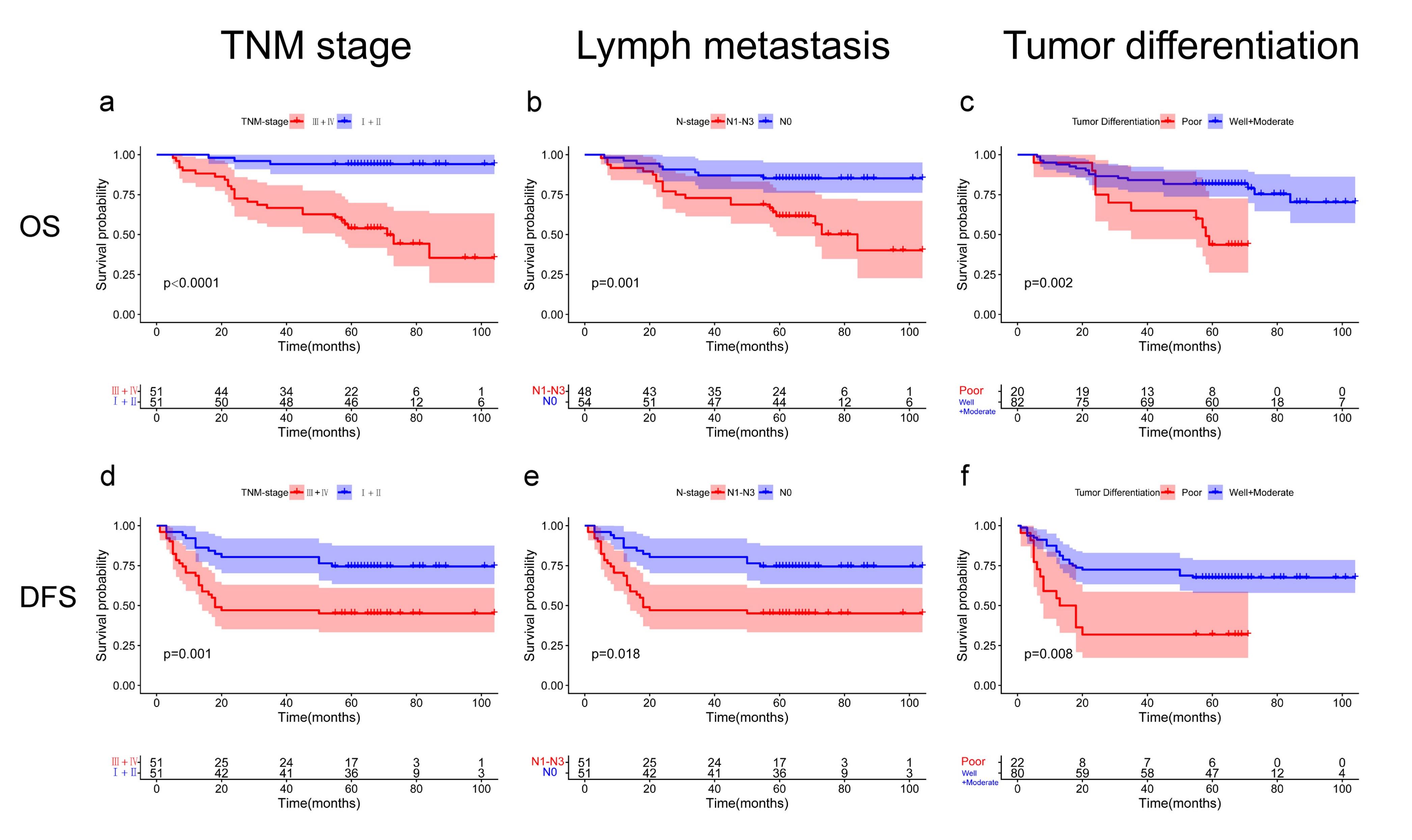

Supplement: Supplementary Figure 3 — Assessment of proportional hazards assumption. [file Image3.jpeg]
