# Supplementary material for: MUC21 is downregulated in oral squamous cell carcinoma and associated with poor prognosis
Source: Front Oncol. 2026 Mar 25;16:1767261. doi: 10.3389/fonc.2026.1767261 (PMC13056625; doi:10.3389/fonc.2026.1767261)
Supplement: Supplementary file 4 [file Table1.docx]

|  | up | down |  | up | down |  | down |
| --- | --- | --- | --- | --- | --- | --- | --- |
| 1 | ADAM12 | A2ML1 | 43 | LAMC2 | GDPD3 | 85 | SH3BGRL2 |
| 2 | ADAMDEC1 | ABI3BP | 44 | MMP1 | GRHL3 | 86 | SLIT3 |
| 3 | AIM2 | ABLIM1 | 45 | MMP10 | HLF | 87 | SLURP1 |
| 4 | AMTN | ABO | 46 | MMP12 | HOPX | 88 | SORT1 |
| 5 | ANGPT2 | ADH7 | 47 | MMP13 | HPGD | 89 | SPINK5 |
| 6 | APOC1 | ALDH3A2 | 48 | MMP3 | ID4 | 90 | SPINK7 |
| 7 | BST2 | ALOX12 | 49 | MMP7 | IL1RN | 91 | SPNS2 |
| 8 | CASP14 | ANGPTL1 | 50 | MMP9 | KAT2B | 92 | STK39 |
| 9 | CCL11 | ANXA1 | 51 | NFE2L3 | KLF8 | 93 | TCP11L2 |
| 10 | CLEC7A | AOX1 | 52 | OAS2 | KLK12 | 94 | TGFBR3 |
| 11 | CTLA4 | APOD | 53 | OAS3 | KLK13 | 95 | TGM3 |
| 12 | CXCL10 | ATP13A4 | 54 | OASL | KRT13 | 96 | TGM5 |
| 13 | CXCL11 | ATP6V1C2 | 55 | OLR1 | KRT3 | 97 | TMPRSS11A |
| 14 | CXCL13 | BARX2 | 56 | PCDH17 | KRT4 | 98 | TMPRSS11B |
| 15 | DCBLD1 | BNIPL | 57 | PDPN | KRT76 | 99 | TMPRSS11D |
| 16 | DDX60 | CD24 | 58 | PI15 | LPIN1 | 100 | UBL3 |
| 17 | DDX60L | CEACAM7 | 59 | PLA2G7 | MAL | 101 | ZNF185 |
| 18 | EPSTI1 | CGNL1 | 60 | PLAUR | MAMDC2 | 102 | ZNF750 |
| 19 | F2RL2 | CILP | 61 | POSTN | MAOB |  |  |
| 20 | FCGR2A | CLDN17 | 62 | PTHLH | MGLL |  |  |
| 21 | FCGR3A | CLDN7 | 63 | PXDN | MGP |  |  |
| 22 | FLNA | CLIC3 | 64 | RSAD2 | MMRN1 |  |  |
| 23 | FN1 | COX7A1 | 65 | SLAMF8 | MPP7 |  |  |
| 24 | GBP1 | CRISP3 | 66 | SP110 | MUC15 |  |  |
| 25 | GBP5 | CRNN | 67 | SPP1 | MUC21 |  |  |
| 26 | GREM1 | CSTB | 68 | TAP1 | MYH11 |  |  |
| 27 | HERC5 | CYP2C18 | 69 | TDO2 | MYO5B |  |  |
| 28 | ICOS | CYP2J2 | 70 | TM4SF19 | NFIA |  |  |
| 29 | IFI27 | CYP3A5 | 71 | TNFRSF12A | NFIX |  |  |
| 30 | IFI35 | CYP4F12 | 72 | TREM1 | NMU |  |  |
| 31 | IFI44 | DAPL1 | 73 | XPR1 | NUCB2 |  |  |
| 32 | IFI44L | DKK4 | 74 |  | PAX9 |  |  |
| 33 | IFIT3 | DPT | 75 |  | PGD |  |  |
| 34 | IL24 | EMP1 | 76 |  | PPL |  |  |
| 35 | IL2RA | EPHX2 | 77 |  | PPP1R3C |  |  |
| 36 | IL7R | ETNK2 | 78 |  | PTGR1 |  |  |
| 37 | INHBA | EXPH5 | 79 |  | RHCG |  |  |
| 38 | ITGA3 | FAM3B | 80 |  | SAMD5 |  |  |
| 39 | ITGAX | FAM3D | 81 |  | SASH1 |  |  |
| 40 | KIF23 | FMO2 | 82 |  | SCEL |  |  |
| 41 | KPNA2 | FUT3 | 83 |  | SCIN |  |  |
| 42 | LAMA3 | GBP6 | 84 |  | SCNN1B |  |  |

**Supplementary Table 1.** The intersection of up and down regulated genes in HTA and GSE34105 and TCGA
