# Supplementary material for: MUC21 is downregulated in oral squamous cell carcinoma and associated with poor prognosis
Source: Front Oncol. 2026 Mar 25;16:1767261. doi: 10.3389/fonc.2026.1767261 (PMC13056625; doi:10.3389/fonc.2026.1767261)
Supplement: Supplementary file 5 [file Table2.docx]

| **Supplementary Table 2.** Association between MUC21 expression and clinicopathologic factors | | | | | |
| --- | --- | --- | --- | --- | --- |
| Variable | No. of Patients（%） | | P |  |  |
| **Age, y** | High（n=） | Low（n=） |  |  |  |
| <60 | 21 | 30 | 0.075 |  |  |
| ≥60 | 30 | 21 |  |  |  |
| **Gender** |  |  |  |  |  |
| Female | 15 | 26 | 0.056 |  |  |
| Male | 36 | 25 |  |  |  |
| **Tumor location** |  |  |  |  |  |
| Tongue | 18 | 24 | 0.188 |  |  |
| Palate | 13 | 4 |  |  |  |
| Buccal | 5 | 5 |  |  |  |
| Floor of mouth | 8 | 8 |  |  |  |
| Gingival | 7 | 10 |  |  |  |
| **Pathologic T classification** |  |  |  |  |  |
| pT1 | 21 | 24 | 0.934 |  |  |
| pT2 | 24 | 22 |  |  |  |
| pT3 | 4 | 3 |  |  |  |
| pT4 | 2 | 2 |  |  |  |
| **Pathologic lymph node metastasis** |  |  |  |  |  |
| Negative (pN0) | 32 | 21 | 0.029 |  |  |
| Positive (pN1-pN3) | 19 | 30 |  |  |  |
| **TNM tumor stage** |  |  |  |  |  |
| I | 13 | 13 | 0.037 |  |  |
| II | 17 | 8 |  |  |  |
| III | 5 | 14 |  |  |  |
| IV | 16 | 16 |  |  |  |
| **Tumor differentiation** |  |  |  |  |  |
| Well | 46 | 34 | 0.010 |  |  |
| Moderate | 4 | 9 |  |  |  |
| Poor | 1 | 8 |  |  |  |

High means high in MUC21 expression, Low means low in MUC21 expression
